# HIV-associated CD8+ encephalitis confirmed by cerebrospinal fluid flow cytometry: first case in Colombia

**DOI:** 10.3389/fmed.2025.1702506

**Published:** 2025-12-02

**Authors:** Nicolas Soto-Moreno, Nicole Pinzon, Natalia Echandia, Omar Rojas-Tabares, Marcela Yanguma, Laura Oviedo, Michael Ariza-Varón

**Affiliations:** 1 Department of Neurology, Universidad del Rosario, Bogotá, Colombia; 2 Faculty of Medicine, Universidad del Rosario, Bogotá, Colombia; 3 Hospital Mayor Mederi, Bogotá, Colombia; 4 Universidad de Ciencias Aplicadas y Ambientales, Bogotá, Colombia; 5 Neurophysiology and Sleep SAS, Bogotá, Colombia; 6 Neurology Unit (Brain Institute), Hospital Universitario Mayor—Mederi, Bogotá, Colombia; 7 Universidad Nacional de Colombia, Bogotá, Colombia; 8 Universidad Internacional de Catalunya, Catalunya, Spain; 9 NeuroUnal Research Group, Bogotá, Colombia

**Keywords:** CD8 positive T lymphocytes, encephalitis, human immunodeficiency virus, flow cytometry, neurology

## Abstract

**Introduction:**

Encephalitis in patients with human immunodeficiency virus (HIV) can be caused by opportunistic infections, immune-mediated processes, or direct viral damage. CD8 + encephalitis is a rare condition. We report the first confirmed case in Colombia, diagnosed by cerebrospinal fluid (CSF) flow cytometry.

**Clinical case:**

A 50-year-old man with a history of liver cirrhosis and HIV, who had suspended antiretroviral treatment 1 month prior to admission, presented to the emergency department with a 2-day history of disorientation, bradyphrenia, dysarthria, and headache. Neurological examination revealed agitation, disorientation, language, memory, and abstraction difficulties, as well as ataxia and generalized chorea. The patient’s CD4 count was 838 cells. Brain magnetic resonance imaging (MRI) showed bilateral asymmetric leukoencephalopathy, and lumbar puncture revealed lymphocytic pleocytosis. After ruling out other differential diagnoses, flow cytometry confirmed the diagnosis of CD8 + encephalitis by identifying 42 cells (59.73% CD8^+^). The patient’s condition improved following the steroid treatment initiation.

**Discussion:**

CD8^+^ T-cell encephalitis is an uncommon immune-mediated disorder in HIV patients, typically occurring when the virus is controlled by antiretroviral therapy. Clinically, it can manifest as global impairment of consciousness, headache and focal symptoms. Diagnosis is typically made via brain biopsy, but imaging and other methods, such as flow cytometry, can be useful. Corticosteroids are the first-line treatment, and prognosis is highly variable.

**Conclusion:**

CD8-mediated encephalitis is a rare condition that requires a complex diagnosis. We present a case of an HIV patient who responded well to corticosteroid therapy without the need for a brain biopsy, confirmed by flow cytometry.

## Introduction

Encephalitis encompasses a group of inflammatory conditions that damage the brain parenchyma, causing focal or diffuse neurological symptoms, including cognitive and behavioral changes (1, 2). These conditions are usually secondary to direct viral invasion, immunitary processes, or opportunistic infections such as cytomegalovirus, cryptococcosis, or tuberculosis in patients with HIV (3). CD8 + encephalitis is a poorly understood immune-mediated process that has recently gained recognition. This condition appears to be independent of CD4 counts because it occurs in patients with a wide range of lymphocyte counts, including normal ones (4, 5). In this article, we present the first case of CD8 + encephalitis confirmed by flow cytometry in Colombia.

## Clinical case

A 50-year-old man with a decade-long history of HIV infection and liver cirrhosis was on antiretroviral treatment (ART) (Abacavir/Lamivudine + Ritonavir/Darunavir) and had suspended his treatment 1 month before admission for 15 days, which he subsequently restarted. The patient presented to the emergency department with a 2-day history of a confusional state, characterized by disorientation, bradyphrenia, dysarthria, and a diffuse, oppressive headache, along with motor restlessness. No other systemic or neurological symptoms were observed. The family denied any prior cognitive symptoms or a diagnosis of HIV-associated cognitive disorder (HAND).

**Figure 1:**
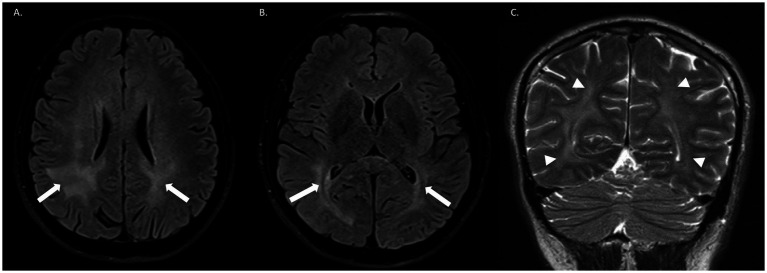
Brain MRI. **(A,B)** Axial FLAIR sequences show bilateral asymmetric involvement of the white matter, predominantly in the occipital regions, particularly periventricular, without cortical involvement or edema (white arrows). **(C)** Coronal T2 sequence demonstrates asymmetric leukoencephalopathy with extensive bilateral subcortical white matter damage, more pronounced in the right hemisphere (white arrowheads).

Neurological examination revealed psychomotor agitation and disorientation to time and person. The patient was unable to comprehend, repeat, or name words, had memory and abstraction impairments, and presented with an ataxic gait and generalized chorea, which was more pronounced in the upper limbs. Initial laboratory studies showed leukocytosis with mild neutrophilia, while electrolytes, coagulation times, and renal, thyroid, and liver functions were within normal limits (Table 1). HIV-induced immunosuppression was confirmed by a reactive 4th-generation ELISA test. Brain MRI revealed an extensive asymmetric leukoencephalopathy pattern, predominantly occipital (Figure 1). The initial differential diagnoses were opportunistic infection and possible progressive multifocal leukoencephalopathy (PML).

**Table 1:** Paraclinical results.

Exams	Results
Complete blood count	Leukocytes: 13,870 cells/μL (neutrophils 82.7%, lymphocytes 10.7%), Hb: 15.1 g/dL, platelets: 300,000/μL.
Hepatic function	Total bilirubin: 0.99 mg/dL (direct 0.25, indirect 0.74); AST: 20 U/L; ALT: 19.2 U/L.
Vitamin deficiency panel	Vitamin B12: 484 μg/L; folate: 18.21 ng/mL; vitamin B1: 19.1 nmol/L; vitamin B6: 3.2 ng/mL; vitamins D and E: within normal limits.
Autoimmune antibodies	ANA: negative; ENA: negative; ANCA: negative.
Drugs of abuse	Negative for all tested substances.
Lumbar puncture #1	Opening pressure: 36 cmH₂O; leukocytes: 117 cells/μL (98.5% lymphocytes); proteins: 82 mg/dL; glucose: 48 mg/dL. Gram stain, cryptococcal antigen, India ink, Ziehl–Neelsen, FilmArray® panel, VDRL, cultures, and PCR for JC virus and Mycobacterium tuberculosis: negative. ADA: 3.58 U/L.
Autoimmune encephalitis (surface antibodies)	NMDA, LGI1, CASPR2, AMPA, GABA-A, GABA-B: all negative.
Antithyroid antibodies	Anti-TPO: negative; anti-TG: negative.
Imaging results	Chest CT scan: calcified granuloma. Brain MRI: bilateral asymmetric involvement of the white matter, predominantly in the occipital regions.
Lumbar puncture #2	Opening pressure: 8 cmH₂O; leukocytes: 117 cells/μL (98.5% lymphocytes); proteins: 82 mg/dL; glucose: 48 mg/dL. CSF PCR for JC virus: negative.
Lumbar puncture #3	Opening pressure: 12 cmH₂O; leukocytes: 198 cells/μL (95.4% lymphocytes); proteins: 77 mg/dL; glucose: 43 mg/dL. HIV CSF viral load: 259 copies/mL.
CSF flow cytometry	CD4⁺CD8⁻: 22.2%; CD4⁺CD8⁺: 6.36%; CD8⁺CD4⁻: 59.73%; CD4/CD8 ratio: 0.37. No atypical lymphoid populations or pathological cells were identified, and no evidence of peripheral blood contamination was observed.
Lumbar puncture #4	Opening pressure: 10 cmH₂O; leukocytes: 6 cells/μL (100% lymphocytes); proteins: 48 mg/dL; glucose: 55 mg/dL (Final lumbar puncture)
Renal function	Creatinine: 0.74 mg/dL; BUN: 12.19 mg/dL; Na: 130 mEq/L; K: 3.55 mEq/L; Cl: 98.9 mEq/L; Ca: 9.33 mg/dL.
HIV serology	Fourth-generation ELISA: reactive (13.0+); viral load in serum: 205 copies/mL.
Infectious serology results	VDRL: non-reactive; hepatitis B surface antigen: negative; anti-HCV: positive; HCV RNA and viral load: negative.
Cell count report	CD4: 838 cells/μL; CD8: 967 cells/μL; CD4/CD8 ratio: 0.87.
Metabolic studies	TSH: 0.390 mUI/L; T4L: 1.24 ng/dL.
Autoimmune encephalitis (intracellular)	Titin, SOX1, Hu, Yo, Ri, Ma2, CV2, amphiphysin: all negative.
Other studies	PT: 11.4 s; PTT: 34.2 s; INR: 1.07; amylase: 103.4 U/L.

The most relevant laboratory findings obtained during hospitalization are presented, particularly those used for the differential diagnosis with infectious, metabolic, autoimmune, or neoplastic entities that could present with encephalitis.

A lumbar puncture revealed an elevated opening pressure of 36 cm H2O, with a cytochemical analysis of CSF showing 117 leukocytes (98.5% lymphocytes), a protein level of 82 mg/dL, and a glucose level of 48 mg/dL (glucose ratio 0.44). Gram stain, Cryptococcus antigen, India ink stain, Ziehl-Neelsen stain, PCR for *Mycobacterium tuberculosis*, Film Array, VDRL, and culture were all negative, and ADA was 3.58 U/L. The patient reported partial improvement of symptoms, particularly headache, following the procedure, and no empirical treatment was initiated. Neuropsychological evaluation confirmed a mild multidomain neurocognitive disorder with predominant executive dysfunction.

Serum studies identified positive antibodies against hepatitis C, which had been treated years earlier, and the viral load was negative, ruling out reinfection. Tests for hepatitis B, drug abuse, thyroid function and deficiency studies (Vitamins B1, B6, B12, folic acid, D, and E) were normal (Table 1). An autoimmune encephalitis panel by immunoblot was performed on serum and CSF, including surface antibodies (NMDA, LGI-1, CASPR2, AMPA, GABA-A, and GABA-B) and intracellular antibodies (Titin, SOX-1, Hu, Yo, Ri, Ma-2, CV-2, and amphiphysin) with negative result. Steroid-responsive encephalopathy with associated thyroiditis (SREAT) was also excluded, and antithyroid antibodies, including antithyroid peroxidase (TPO) and antithyroglobulin (Tg), were negative. Other tests for autoimmune conditions, including antinuclear antibodies, extractable antibodies, and antineutrophil cytoplasmic antibodies, were also performed with negative results (Table 1).

Subsequently, the immunological report showed a CD4 count of 838 cells, a CD8 count of 967 cells, a serum CD4/CD8 ratio of 0.87, and a viral load of 205 copies. Given these results and a negative CSF PCR result for JC virus, the probability of PML was considered low. A chest CT scan showed a calcified granuloma, but the probability of central nervous system (CNS) tuberculosis was also considered low because of the patient’s clinical stability and negative CSF findings, in addition to a Thwaites score of 4 and a Marais score of 9 points. Therefore, empirical treatment for tuberculosis was not initiated. Other opportunistic conditions were deemed unlikely based on CSF results and CD4 levels. Follow-up lumbar puncture demonstrated improved opening pressure (8 cm H2O) but persistent inflammatory signs. Following the lumbar puncture, the patient demonstrated a partial improvement in symptoms, particularly headache, likely secondary to the decrease in intracranial pressure. Additional infectious studies were negative, and the ADA control was 4.69 U/L. The follow-up MRI showed no changes, leading to the differential diagnosis of HIV and CD8 + encephalitis. The patient did not meet the criteria for immune reconstitution inflammatory syndrome (IRIS).

**Figure 2:**
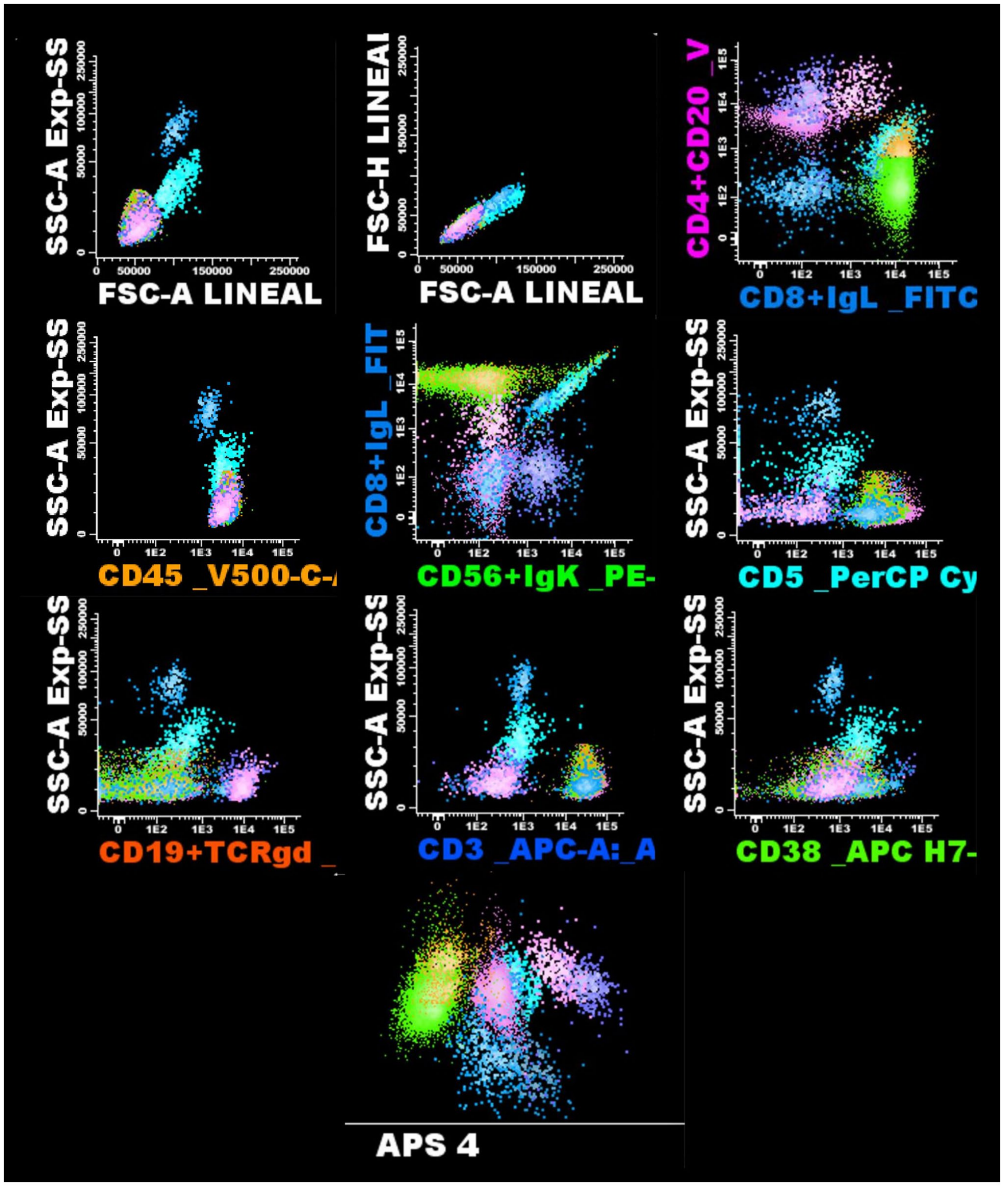
Flow cytometry of cerebrospinal fluid. The gating strategy used for the analysis of lymphocyte populations in the patient’s sample is shown. Sequential evaluation of the lymphocyte subpopulations and the most relevant spots are displayed.

Therefore, we decided to expand the CSF studies. A third CSF sample showed increased pleocytosis (197 leukocytes), persistent hyperproteinorrachia (77 mg/dL), and similar glucose levels (43 mg/dL). We performed flow cytometry with the following immunophenotypic markers: CD20-CD4/CD45/CD8-IgGLambda/CD 56-Igkappa/CD5/CD19-TCRg/d/ CD3 and CD38 with a proper gating strategy (Figure 2), which reported 42 cells/μL including lymphoid cells (96.23%), most of which were T lymphocytes (90.21%), differentiated as CD4^+^CD8^−^ (22.2%), CD4^+^CD8^+^ (6.36%), and CD8^+^CD4^−^ (59.73%). The CSF CD4/CD8 ratio was 0.37. No atypical or pathological cells were identified, and no evidence of peripheral blood contamination was found. The CSF viral load was 259 copies/mL, similar to the blood result, indicating a possible viral escape.

**Figure 3:**
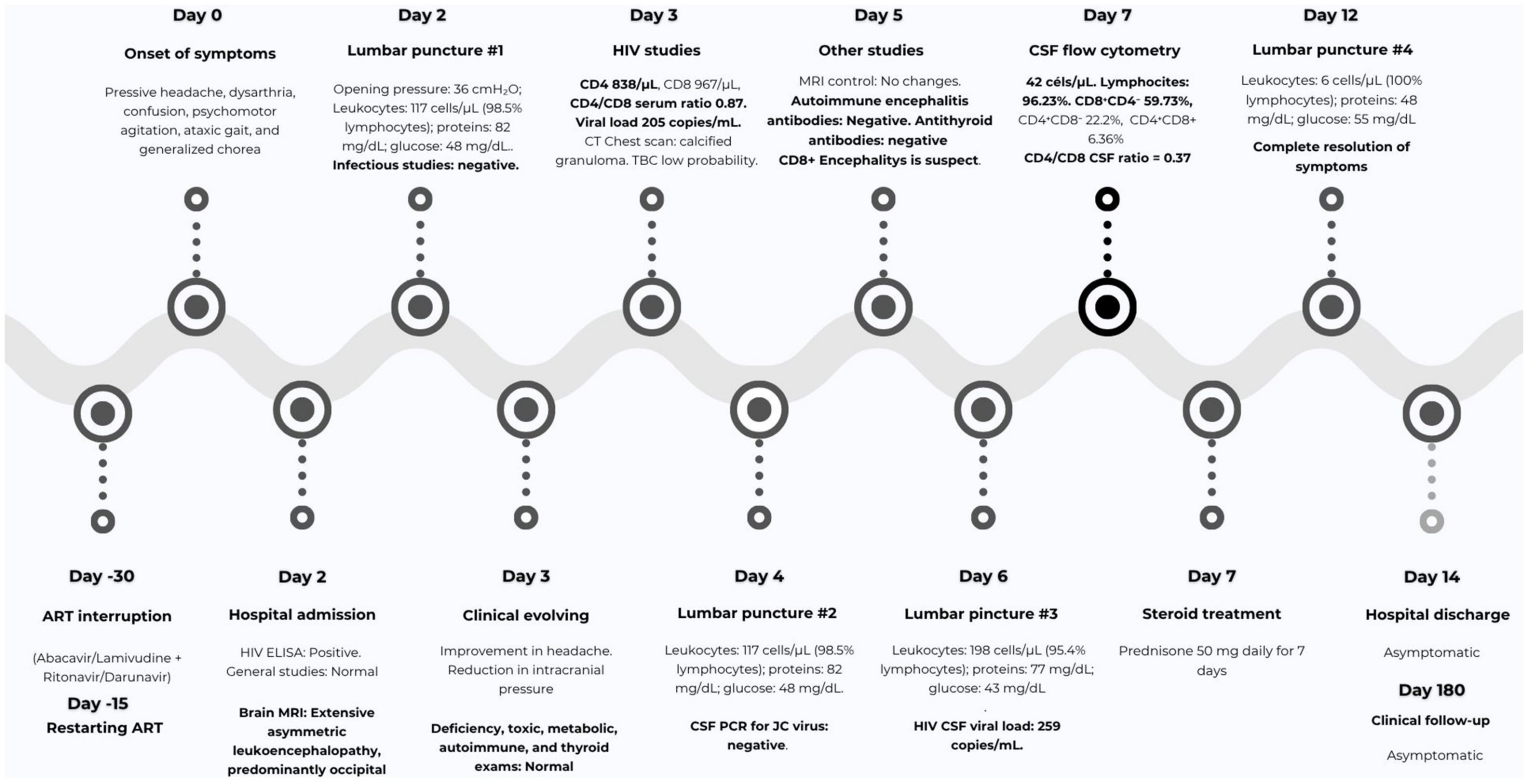
Timeline of the patient's clinical course. The most relevant milestones in the patient’s hospital course are presented, emphasizing the onset of neurological symptoms, the execution of major diagnostic procedures (including lumbar punctures), and the initiation of corticosteroid therapy.

Based on the flow cytometry results, brain biopsy was considered unnecessary. A diagnosis of CD8 + encephalitis was confirmed, which was likely triggered by the temporary suspension and restart of antiretroviral therapy. Treatment with oral corticosteroids (prednisolone 50 mg daily for 7 days) was initiated, and the patient exhibited a noticeable clinical improvement on the fifth day of treatment. Following this management, the patient experienced a complete resolution of symptoms and improved cognitive function. A new lumbar puncture showed improvement in inflammatory signs (Table 1). The patient remains asymptomatic after 6 months of follow-up. The most important events of the clinical case, organized in a timeline, are represented in Figure 3.

## Discussion

Encephalitis is a neurologically significant condition associated with high mortality and long-term disability. In most cases, the etiology remains unknown despite exhaustive investigation (1). Viral infections are the most frequent known cause, followed by autoimmune encephalitis (e.g., anti-NMDA, LGI1, GABA, and AMPAR). Other recognized etiologies include bacterial or fungal agents, as well as toxic, metabolic, and systemic autoimmune conditions such as systemic lupus erythematosus, Sjögren’s syndrome, and sarcoidosis (1, 2). These conditions present a global challenge due to their high morbidity and mortality, making early diagnosis and prompt treatment critical for improving patient outcomes (1, 6).

Multiple causes of encephalitis exist in HIV-immunosuppressed patients, including opportunistic infections, inflammatory/autoimmune etiologies, and direct viral mechanisms like HIV encephalitis and HAND (3). Opportunistic infections include viral agents (e.g., cytomegalovirus, herpes zoster, and JC virus), bacteria (e.g., neurosyphilis), and parasites (e.g., *Toxoplasma gondii*) and fungi (e.g., *Cryptococcus* spp.) (6, 7). Autoimmune causes, such as autoantibody-mediated encephalitis, can be triggered by prior infections in patients with HIV (3, 4). Encephalitis mediated by inflammatory changes secondary to antiretroviral treatment complications, such as IRIS, and CD8 + encephalitis are less common (4, 8).

CD8 + T-cell encephalitis is an immune-mediated disorder caused by dysregulated inflammatory responses of T-lymphocytes in patients with HIV (4). The pathophysiology of the disease is characterized by diffuse perivascular and parenchymal infiltration of CD8 + T cells, microglial activation, and reactive astrocytes. The absence of multinucleated giant cells is also a recognized marker of this condition (9, 10). The proposed mechanism for CD8 + encephalitis is an immunological imbalance within the CNS where the CD8 + T-cell response against HIV “overshoots its objective,” causing local damage. Despite the massive infiltration of CD8 + cells, their low efficiency in eliminating infected resident cells such as macrophages means that, instead of controlling the virus, they sustain chronic inflammation and *cause* neurotoxicity by releasing cytokines (11). This dysfunction, characterized by CD8 + cells exhibiting high activation but low cytolytic capacity during chronic stages, leads to an autonomous and sustained inflammation within the cerebral tissue that persists even with minimal evidence of active viral infection (11, 12). HIV viral escape into the CNS is a significant triggering factor, which is often associated with the temporary suspension of ART (8).

This clinical entity was first described in 2004 by *Miller et al.*, who reported on two cases of encephalopathy in patients with HIV. In these patients, postmortem histology confirmed marked brain inflammation caused by CD8 + T-cells (5). This condition can occur regardless of a patient’s disease control and has been observed even in patients who are clinically stable on ART (4). CD8 lymphocytes have also been implicated in other neurological conditions, such as Rasmussen’s encephalitis, limbic encephalitis, and *Toxoplasma gondii*-induced encephalitis (4, 13).

CD8 + T-cell encephalitis is a rare condition, with relatively few cases described in the literature and a female predominance (4). While most reported cases of HIV-associated CD8 + T-cell encephalitis have involved female and Black African patients, our report of a Hispanic male patient broadens the known demographic spectrum of this condition. This observation suggests that the disorder may occur across a wider range of populations than previously recognized, emphasizing the need for awareness beyond traditionally reported demographic groups (7, 8).

The largest published series, comprising 53 patients, was reported by Lucas et al. (8) in a study from England and Wales with an autopsy rate of nearly 20% (2000–2019), among 500 deaths in HIV-positive adults, one fatal case was pathologically diagnosed annually. Santana et al. (14) also previously documented a systematic review of the most relevant cases. A significant challenge in diagnosis is that autopsies for medical deaths are rare in places like Africa and Latin America, suggesting that CD8 + T-cell encephalitis deaths are likely not recognized or reported in these regions (10, 15).

The clinical characteristics vary, but the disease typically has an acute or subacute course. The most common symptoms include encephalopathy, headache, ataxia, confusion, and seizures (9, 14). Symptom frequency may differ by sex. Headaches are more common in women (62% vs. 27% in men), whereas cognitive impairment is more frequent in the male population (41% vs. 12% in women). This impairment can range from confusion to coma (5, 8). These symptoms are similar to those in our case, except for chorea, which has not been described as a predominant symptom.

The described patients have an average CD4 count of 315 cells/microliter and an average viral load of 600 copies, although this is highly variable, and the condition can occur in patients with negative viral loads (5, 16). The condition most commonly occurs in patients without severe immunosuppression and even in those with relatively normal CD4 counts, as observed in our patient. The suspension of ART and the presence of viral escape, evidenced by a positive viral load in the CSF, are the most relevant factors for the development of this disease (4, 8).

Diagnosis is based on clinical findings MRI, CSF analysis, and histopathological results (14, 17, 18). A definitive diagnosis can only be made through a brain biopsy, which is why many cases are confirmed postmortem (15, 16). Histopathological examination has a 96% diagnostic yield, showing parenchymal and perivascular inflammation with CD8 lymphocyte and macrophage infiltration. However, this test has only been performed in 40% of suspected cases, and no specific *in vivo* biomarkers are currently available (4, 8, 19, 20).

CSF findings show lymphocytic pleocytosis in 74% of cases, with an average of 20–21 cells/mL and a predominance of CD8 + cells with a reversal of the CD4/CD8 ratio in 90% of patients. Other characteristics include moderate hyperproteinorrachia and a positive CSF HIV viral load, which is often higher than the serum viral load, indicating viral escape (10, 19, 20). Our patient’s CSF profile, including the reversal ratio and positive viral load, aligns with these findings. In this case, the serum CD4/CD8 ratio was 0.87, whereas it was 0.37 in the CSF. This indicates a clear inversion, with a predominance of CD8 + T cells in the CNS. This finding suggests compartmentalized activation and proliferation of these cells, whose overexpression likely contributed to CNS damage, particularly affecting the white matter, and the clinical manifestations of encephalitis observed in the patient.

Notably, the inversion of the CD4/CD8 ratio in the CSF provided critical diagnostic value, supporting the diagnosis through immunophenotypic analysis by flow cytometry, and highlighting its utility in identifying CNS-specific immune dysregulation. The use of flow cytometry for diagnosis has been little explored, with no solid evidence or inclusion in diagnostic criteria. However, this case underscores the potential diagnostic value of CSF flow cytometry as a less invasive and accessible method to identify CD8 + T-cell predominance, particularly in scenarios where brain biopsy is not feasible. This approach highlights a novel and clinically useful diagnostic pathway that may allow earlier recognition and treatment of HIV-associated CD8 + T-cell encephalitis.

Typical MRI findings are present in 77% of cases and are characterized by symmetrical, confluent, bilateral hyperintense lesions with slight contrast enhancement, predominantly in the white matter. Occasional enhancement may also be observed at the cortical level and in the basal ganglia. Findings are considered atypical when only bilateral white matter lesions without the described enhancement are observed (8, 13, 18).

The literature recommends ruling out other potential causes of encephalitis that could explain the patient’s symptoms, clinical findings, and CSF changes. These include HIV encephalitis, HAND, JC virus infection (LMP), cytomegalovirus (CMV), varicella zoster virus (VZV), enterovirus, herpes simplex virus (HSV), bacterial meningitis, and autoimmune encephalitis (9, 15, 16). We methodically ruled out key differential diagnoses pertinent to the presentation of our patient. Specifically, HAND was excluded, primarily due to the CSF analysis, which typically shows detectable viral load but lacks the consistent pleocytosis observed in our case. Primary CNS lymphoma was dismissed based on the absence of atypical lymphocytes in the CSF cytology, coupled with our patient’s favorable clinical response and progressive improvement. Primary and secondary CNS vasculitis were excluded based on a negative comprehensive autoimmunity panel and distinct neuroradiological findings, which did not reveal the typical pattern of cerebral ischemia, hemorrhage, or cortical involvement associated with vasculitic processes. Finally, IRIS was excluded because there was no occurrence of a paradoxical deterioration and did not exist an opportunistic CNS infection. Studies on autoimmune encephalitis and SREAT were negative. In some instances, CD8 + encephalitis may be confused with primary CNS tumors, or Posterior Reversible Encephalopathy Syndrome (21–23).

In the acute phase of the disease, the treatment of choice is high-dose corticosteroids, with both intravenous methylprednisolone and oral prednisolone being described (13, 15). Some reports have described the need for chronic corticosteroid therapy and other immunomodulators, such as mycophenolate, due to symptom recurrence (9, 20). Prompt initiation of therapy has been shown to result in symptomatic improvement and decreased mortality (8, 14). Optimizing antiretroviral treatment is essential, especially in cases with evidence of viral escape into the CNS. The preferred approach involves tailoring therapy based on resistance testing and viral mutations in the CSF, with integrase inhibitors such as dolutegravir being preferred (4, 5, 10, 22). Our patient responded optimally to oral steroid treatment, which is consistent with the literature published to date. CD8 + encephalitis has a variable prognosis, with outcomes ranging from complete recovery to irreversible sequelae and even death (4, 8, 10).

## Conclusion

CD8 + encephalitis is a rare and underdiagnosed condition that causes cognitive and behavioral changes in patients with HIV, even those with normal CD4 counts and those on antiretroviral therapy. The diagnosis is complex and usually requires a brain biopsy. However, as demonstrated in this case, flow cytometry to identify CD8 lymphocyte infiltration may be a valuable diagnostic tool. The treatment of choice is corticosteroids. This is the first confirmed case of this condition in Colombia.

## Data Availability

The raw data supporting the conclusions of this article will be made available by the authors, without undue reservation.

## References

[1] Alam AM Easton A Nicholson TR Irani SR Davies NWS Solomon T . Encephalitis: diagnosis, management and recent advances in the field of encephalitides. Postgrad Med J. (2023) 99:815–25. doi: 10.1136/postgradmedj-2022-141812, PMID: 37490360

[2] Venkatesan A. Encephalitis and brain abscess. Continuum (Minneap Minn). (2021) 27:855–86. doi: 10.1212/CON.0000000000001006, PMID: 34623096

[3] Grill MF. Neurologic complications of human immunodeficiency virus. Continuum (Minneap Minn). (2021) 27:963–91. doi: 10.1212/CON.0000000000001035, PMID: 34623100

[4] Shenoy A Marwaha PK Worku DA. CD8 encephalitis in HIV: a review of this emerging entity. J Clin Med. (2023) 12:770. doi: 10.3390/jcm12030770 36769419 PMC9917721

[5] Miller RF Isaacson PG Hall-Craggs M Lucas S Gray F Scaravilli F . Cerebral CD8+ lymphocytosis in HIV-1 infected patients with immune restoration induced by HAART. Acta Neuropathol. (2004) 108:17–23. doi: 10.1007/s00401-004-0852-0, PMID: 15085359

[6] Tunkel AR Glaser CA Bloch KC Sejvar JJ Marra CM Roos KL . The management of encephalitis: clinical practice guidelines by the Infectious Diseases Society of America. Clin Infect Dis. (2008) 47:303–27. doi: 10.1086/589747, PMID: 18582201

[7] Reimer-Mcatee M Ramirez D Mcatee C Granillo A Hasbun R. Encephalitis in HIV-infected adults in the antiretroviral therapy era. J Neurol. (2023) 270:3914–33. doi: 10.1007/s00415-023-11735-w, PMID: 37115358 PMC11332430

[8] Lucas SB Wong KT Nightingale S Miller RF. HIV-associated CD8 encephalitis: a UK case series and review of Histopathologically confirmed cases. Front Neurol. (2021) 12:628296. doi: 10.3389/fneur.2021.628296, PMID: 33868143 PMC8047670

[9] Sharma R Spradley T Campbell M Biyani S Singhal P Elkhider H . CD8 encephalitis: a diagnostic dilemma. Diagnostics (Basel). (2022) 12:2687. doi: 10.3390/diagnostics12112687, PMID: 36359530 PMC9689240

[10] Mirgh SP Mishra VA Harbada RK Sorabjee JS. Knowing the unknown—CD8 encephalitis: a novel form of HIV-associated neurocognitive disorder. Neurol India. (2019) 67:261–4. doi: 10.4103/0028-3886.253630, PMID: 30860129

[11] Subra C Trautmann L. Role of T lymphocytes in HIV neuropathogenesis. Curr HIV/AIDS Rep. (2019) 16:236–43. doi: 10.1007/s11904-019-00445-6, PMID: 31062168 PMC6579685

[12] Kessing CF Spudich S Valcour V Cartwright P Chalermchai T Fletcher JLK . High number of activated CD8+ T cells targeting HIV antigens are present in cerebrospinal fluid in acute HIV infection. J Acquir Immune Defic Syndr. (2017) 75:108–17. doi: 10.1097/QAI.0000000000001301, PMID: 28177966 PMC5388590

[13] Morioka H Yanagisawa N Sasaki S Sekiya N Suganuma A Imamura A . CD8 encephalitis caused by persistently detectable drug-resistant HIV. Intern Med. (2016) 55:1383–6. doi: 10.2169/internalmedicine.55.5783, PMID: 27181553

[14] Santana LM Valadares EA Ferreira-Júnior CU Santos MF Albergaria BH Rosa-Júnior M. CD8 + T-lymphocyte encephalitis: a systematic review. AIDS Rev. (2020) 22:112–22. doi: 10.24875/AIDSRev.20000132, PMID: 32180590

[15] Cheema A Mathias K Bui C Dunham SR Goodman JC El Sahly HM. CD8 encephalitis in a treatment-naive and a virologically suppressed patient with HIV. Can J Neurol Sci. (2019) 46:773–5. doi: 10.1017/cjn.2019.288, PMID: 31466541

[16] Lescure FX Moulignier A Savatovsky J Amiel C Carcelain G Molina JM . CD8 encephalitis in HIV-infected patients receiving cART: a treatable entity. Clin Infect Dis. (2013) 57:101–8. doi: 10.1093/cid/cit175, PMID: 23515205

[17] Kerr C Adle-Biassette H Moloney PB Hutchinson S Cryan JB Clarke S . CD8 encephalitis with CSF EBV viraemia and HIV drug resistance, a case series. Brain Behav Immun Health. (2020) 9:100164. doi: 10.1016/j.bbih.2020.100164, PMID: 34589901 PMC8474158

[18] Manesh A Barnabas R Karthick R Chacko G Kannangai R Varghese GM. HIV-mediated CD8 encephalitis: an under recognised entity. Int J Infect Dis. (2016) 45:264. doi: 10.1016/j.ijid.2016.02.588

[19] Salam S Mihalova T Ustianowski A McKee D Siripurapu R. Relapsing CD8+ encephalitis-looking for a solution. BMJ Case Rep. (2016) 2016:bcr2016214961. doi: 10.1136/bcr-2016-214961, PMID: 27335359 PMC4932356

[20] Heng A Burkett A Akkol S McCay M Krishnam SP Wick N . Case report of CD8 encephalitis in a person living with HIV. Neurology. (2024) 103:S141. doi: 10.1212/01.wnl.0001051992.96086.bb

[21] Wood AC Parker R Allinson K Scoffings D. CD8 encephalitis presenting as autoimmune encephalitis in HIV-1 infection. BMJ Case Rep. (2022) 15:e246290. doi: 10.1136/bcr-2021-246290, PMID: 35459644 PMC9036170

[22] Ishiguro M Ueno Y Ishiguro Y Takanashi M Murai K Taieb G . CD8+ T-cell encephalitis mimicking PRES in AIDS: a case report. BMC Neurol. (2020) 20:179. doi: 10.1186/s12883-020-01756-7, PMID: 32397957 PMC7216593

[23] Moulignier A Savatovsky J Polivka M Boutboul D Depaz R Lescure FX. CD8 T lymphocytes encephalitis mimicking brain tumor in HIV-1 infection. J Neurovirol. (2013) 19:606–9. doi: 10.1007/s13365-013-0217-3, PMID: 24277438

